# The cerebrospinal fluid immune cell landscape in animal models of multiple sclerosis

**DOI:** 10.3389/fnmol.2023.1143498

**Published:** 2023-04-12

**Authors:** Gregory F. Wu

**Affiliations:** ^1^Departments of Neurology and Pathology & Immunology, Washington University School of Medicine, St. Louis, MO, United States; ^2^Neurology Service, VA St. Louis Health Care System, St. Louis, MO, United States

**Keywords:** experimental autoimmune encephalomyelitis, multiple sclerosis, animal model, cerebrospinal fluid, neuroimmunology

## Abstract

The fluid compartment surrounding the central nervous system (CNS) is a unique source of immune cells capable of reflecting the pathophysiology of neurologic diseases. While human clinical and experimental studies often employ cerebrospinal fluid (CSF) analysis, assessment of CSF in animal models of disease are wholly uncommon, particularly in examining the cellular component. Barriers to routine assessment of CSF in animal models of multiple sclerosis (MS) include limited sample volume, blood contamination, and lack of feasible longitudinal approaches. The few studies characterizing CSF immune cells in animal models of MS are largely outdated, but recent work employing transcriptomics have been used to explore new concepts in CNS inflammation and MS. Absence of extensive CSF data from rodent and other systems has curbed the overall impact of experimental models of MS. Future approaches, including examination of CSF myeloid subsets, single cell transcriptomics incorporating antigen receptor sequencing, and use of diverse animal models, may serve to overcome current limitations and provide critical insights into the pathogenesis of, and therapeutic developments for, MS.

## 1. Introduction

*The sciences do not try to explain, they hardly even try to interpret, they mainly make models. By a model [it is meant a] construct which, with the addition of certain verbal interpretations, describes observed phenomena. The justification of such a mathematical construct is solely and precisely that it is expected to work – that is, correctly to describe phenomena from a reasonably wide area. Furthermore, it must satisfy certain esthetic criteria – that is, in relation to how much it describes, it must be rather simple.*

— John von Neumann (Von Neumann, 1955)

### 1.1. Rationale for examining spinal fluid in multiple sclerosis

A lumbar puncture, or spinal tap, has routinely been utilized diagnostically and/or therapeutically since its development as a modern medical procedure in the late 19th century (Marsala et al., 2015). Examination of leukocytes contained within cerebrospinal fluid (CSF) has provided opportunities to glean basic pathophysiologic traits of neurologic diseases including meningitis, encephalitis, and autoimmune conditions such as multiple sclerosis (MS) (Goetz, 2007). While routine studies of the cellular constituents of CSF employed in the clinical setting based on cytochemical analysis remain elementary (Shahan et al., 2021), specialized analyses suggest more detailed characterization of the cellular component is meaningful for understanding the pathogenesis of MS. For example, intrathecal accumulation of oligoclonal bands (OCBs), a diagnostic and prognostic tool for MS (Avasarala et al., 2001; Freedman et al., 2005), has spurred efforts toward identifying the nature of CSF plasmablasts abundant in MS (Alvermann et al., 2014) as well as defining their antigen-specificity (Eggers et al., 2017; Lanz et al., 2022).

The compartmentalization of inflammatory responses in the CNS establishes a *de facto* limitation of focusing solely on the blood to study the initiation and propagation of inflammatory demyelination in MS. Significant differences in lymphoid and myeloid composition and phenotypic traits between CSF and blood have been described in MS. For example, the ratio of CD4 and CD8 T cells is distorted between compartments in MS, with flow cytometric studies demonstrating a higher CD4/CD8 ratio in the CSF (Kölmel and Sudau, 1988). More contemporary characterization by single cell RNA sequencing (scRNA-seq) has further defined disparities in immune cell populations between compartments, including unique functional traits of B cells in the CSF of MS patients (Ramesh et al., 2020). In terms of myeloid populations, various subsets of monocytes predominate in the blood of MS patients (Esaulova et al., 2020), while microglia-like cells are exclusively isolated from the CSF (Farhadian et al., 2018; Esaulova et al., 2020). The microenvironment of the CSF may impart distinctive cues on leukocytes such as myeloid cells that dictate tissue-specific phenotypes (Pashenkov et al., 2002), suggesting how cells within the CNS compartment may be influenced during inflammatory changes occurring in MS. Thus, analysis of immune cells from the CSF provides an exclusive assessment of the CNS compartment not available by interrogation of peripheral blood.

Critically, cells within the CSF reflect immune activity proximal to the tissues damaged by inflammation in MS, namely the optic nerves, brain, and spinal cord. CSF studies enabled by a relatively simple lumbar puncture circumvent more complicated and risk-prone procedures such as tissue biopsy to garner aspects of immune processes within the target organ. Moreover, it is now more clear than ever that CNS borders and barriers play an integral role in maintaining tissue-specific immunity and regulating inflammatory responses (Alves de Lima et al., 2020; Buckley and McGavern, 2022). Since CSF is directly in contact with the meninges, choroid plexus and perivascular spaces, cellular composition within the CSF offers a direct representation of what can go awry at the borders in CNS autoimmunity. Indeed, changes specific to progressive MS such as the formation of ectopic lymphoid tissue (ELT) (Serafini et al., 2004) suggest how important CSF cellular markers are to capturing the spectrum of inflammatory features in MS throughout different diseases states, particularly within the meninges. It appears that border associated macrophages (BAMs) exist within the CSF (Ostkamp et al., 2022), but whether they circulate within the CSF and/or represent the tissue state of myeloid cells within the parenchyma remains to be determined. Capturing features of meningeal and other barrier changes in MS will likely be important in discovering additional footholds for therapeutic intervention.

### 1.2. Rationale for using animal models in the study of MS

What is achieved by using animal models to study MS? As denoted by the celebrated applied mathematician John von Neumann, modeling serves as the main tool for scientific pursuits to not so much identify the reasons for phenomena in nature, but rather generate elements to describe mechanisms underpinning these phenomena. By making precise observations and generating descriptive interpretations from models, a refinement of various components of observed phenomena has produced much of the current understanding of the pathophysiology of MS and related diseases (Constantinescu et al., 2011; Rangachari and Kuchroo, 2013). In addition to advantages such as animal genetics, speed of reproduction, ethical issues, and potential simplification of systems supporting the use of animal models in research (Chesselet and Carmichael, 2012; Mukherjee et al., 2022), the translational possibilities of models such as experimental autoimmune encephalomyelitis (EAE) has solidified the reliance on animal experimentation to drive therapeutic innovation for MS.

There are numerous drawbacks to reliance on EAE and other animal models as complete and faithful representations of all aspects of MS. Indeed, multiple facets of MS pathogenesis have not been adequately captured by modeling (Lassmann and Bradl, 2017). For example, the relevance of cytotoxic CD8 T cells in MS has been underestimated in most EAE models (Goverman et al., 2005; Mars et al., 2011), and no animal model exists that exemplifies the collection of key traits of progressive MS (Lassmann and Bradl, 2017; Baker et al., 2019).

Nevertheless, strategic use of animals for experimental models of MS affords potential value in many respects. Regarding the reductionistic aspect of EAE and other commonly used models for MS, particularly the murine systems, it has been argued that “…each model recapitulates a small piece of the human disease” (Van Epps, 2005). Hence, using EAE as a simplified strategy to optimize mechanistic understanding of immune cells within the CNS during disease over time and in different contexts has, and promises to remain, a valuable tool for understanding MS. By extension, analyses of immune cell composition and phenotypes within the CSF is vital to maximizing understanding of the immuno-pathophysiology of MS and developing the insight required for even further efficacious therapies.

### 1.3. Challenges of CSF cellular analysis in animal models of MS

With the advent of transgenic technology, rodents became the favored model organism for studying MS, with a preponderance of experimental reports on the immunologic aspects of MS derived from EAE in mice (Rangachari and Kuchroo, 2013). Adult mice harbor approximately 40 μL of CSF at any given time, with an average CSF production rate of 0.38 ± 0.02 μL/min (Oshio et al., 2005). Thus, one major challenge in exploring immune cell traits within the CSF during murine EAE is the limited volume of fluid available for study. Typical procurement of CSF from mice is through cisternal puncture and garners a small volume (5–7 μL). However, certain protocols offer an opportunity to double (Lim et al., 2018) or triple this volume (Maia et al., 2013), the latter due to use of repeated sampling. Blood contamination from the isolation technique is also of primary concern given the location of multiple vessels near the cisterna magna (You et al., 2005; Lim et al., 2018). While lateral ventricle sampling does not increase the acquired volume or reduce potential for blood contamination (McIntyre et al., 2019), sampling through implanted ventricular catheters (Herzog et al., 2021) could facilitate longitudinal studies and may improve acquisition for successful studies on CSF immune cells in the future, as could modified procedures to ensure survival through repeated acquisition *via* the cisterna magna (Han et al., 2022).

## 2. CSF immune cells in animal models of MS: The status quo

Current knowledge regarding the immune cell composition within CSF from animal models of MS is limited. Somewhat surprisingly, the majority of studies examining the cellular constituents of CSF in EAE were undertaken decades ago. Almost 20 some years after the development of EAE (Van Epps, 2005) a series of reports in rhesus monkeys included a brief quantification of white blood cells within the CSF (Kabat et al., 1951), with a general pleocytosis being evident in diseased animals. Of note, there did not appear to be a correlation between CSF white cells and total protein or immunoglobulin. At that time, modern analytical tools were not available, but use of animal models to pursue hypothesis testing and concept development in MS pathogenesis research nevertheless persisted (Baxter, 2007).

Unsurprisingly, the majority of studies examining CSF in animal models of MS involve larger species such as the rat or guinea pig. Given the intense dedication early on during MS research to the concept that T cells are central to the pathogenesis of MS (Martin et al., 1992), CSF studies similarly tended to focus on T cells. In the late 1970s, a group at the University of Pennsylvania characterized basic CSF cellular features in guinea pig EAE. At the onset of neurologic impairment, a 30-fold increase in white cells was observed in the CSF, noted to be typically on the order of 100 μL per specimen. Using erythrocyte rosette testing and cytochemistry, they concluded the vast majority of CSF leukocytes were T cells (Wilkerson et al., 1978). In the late 1980s, Rumsby and colleagues published several reports on the CSF profile in guinea pig EAE induced by immunization with guinea pig spinal cord homogenate emulsified in complete Freund’s adjuvant (CFA) (Suckling et al., 1986, 1987). Analyses were based on immunocytochemical detection of T cells and macrophages over time in this chronic relapsing model of MS. Acquiring what could be over 150 μL of CSF, they observed total CSF leukocytes building alongside disease development, with peak clinical scores corresponding to maximal numbers of CSF leukocytes (Suckling et al., 1986). T cells in an activated state (based on IL-2 receptor staining) were found in similar proportion in both the CSF and meninges (Suckling et al., 1987). The significant relationship between activated T cells in the blood and CSF piqued interest in deciphering movements of T cells between the periphery and CNS during EAE (Suckling et al., 1987). Later, another group explored the trafficking and T cell receptor (TCR) diversity of T cells within the CSF of Lewis rats with EAE. Examination of CSF acquired by cisternal puncture from active EAE induced by immunization with guinea pig MBP required pooled specimens from three animals for analysis. A bias toward Vβ8.2+ T cells was observed in both the CSF as well as spinal cord tissue, and could be detected without any restimulation *ex vivo* (Offner et al., 1993). Based on flow cytometric quantification, T cells first appeared in CSF before clinical deficits developed and then accumulated within the spinal cord at disease onset (Buenafe et al., 1994). By defining the CDR3 sequences of T cells in the CSF and spinal cord parenchyma, it was concluded that “…CSF-derived T cells provide a representative view of CNS events at the onset of EAE.” Defining trafficking of lymphocytes within the CNS compartment during neuroinflammation can involve assessment of CSF along with border tissues and parenchyma for relative comparison of localization and abundance. An attempt was made at correlating various tissue locations of immune cells including CSF during EAE in rats. A minimum of 50 μL of CSF was analyzed from DA rats immunized with guinea pig spinal cord homogenate (Schmitt et al., 2012). In contrast to rats immunized with CFA alone that contained less than 1 cell/μL of CSF, rats with early stages of EAE had an average of over 60 cells/μL of CSF which were comprised of neutrophils and monocytes, but primarily CD3+ T cells. *In situ*, CD45+ cells accumulated within the ventricular system of the forebrain and midbrain early in disease, with subsequent accumulation throughout cisterns and the ventricular system. These results suggest that initial homing of T cells to CNS sites during EAE occurs at certain rostral anatomic regions and utilizes the CSF and CSF-adjacent tissues for trafficking (Schmitt et al., 2012). Functional characterization of CSF T cells was performed in a limited study by Renno et al. (1994) who examined the CSF of SJL mice with EAE. While the volume of CSF obtained per animal was not specified, pooled specimens were used, and an average number of CSF cells was reported. Interestingly, control mice immunized with CFA alone harbored similar numbers of cells in the CSF as naive mice. A four-to five-fold increase in CSF cell count was observed depending on severity of EAE. Production of IL-2 and IFNγ by CSF leukocytes was detected by quantitative PCR and correlated with disease severity, mirroring the relation of cytokine expression in the parenchyma with impairment (Renno et al., 1994). In sum, CSF studies in animal models of MS initially all reinforced the notion that T cells serve as central actors in the pathogenesis of MS.

More contemporary studies have also pursued aspects of T cell-driven disease by employing rat EAE models of MS. T cells detected in the subarachnoid space by intravital imaging exhibit migrational behaviors indicative of antigen-specific interactions (Bartholomäus et al., 2009). Work from this same group extended these observations in part by profiling encephalitogenic T cells specific for MBP in different compartments such as the spinal cord parenchyma, meninges, blood and unspecified quantities of CSF (Schläger et al., 2016). Isolated from the CSF by stereotactic-guided cisternal magna puncture and quantified by flow cytometry, the frequency of MBP-specific T cells in the CSF peak during the course of the disease at the same time as in the meninges, albeit in lower numbers. Comparing MBP-specific T cells isolated from each compartment using bulk RNA sequencing, activation markers were found to be more pronounced in antigen-specific T cells in the meninges and parenchyma than in the CSF, suggesting that circulation through the fluid compartment of the CNS could serve as a staging area or location of lymphocyte quiescence. Yet a heroic experiment testing this concept was able to demonstrate that MBP-specific T cells re-isolated from the CSF were still able to induce EAE upon re-activation prior to transfer into naive rats (Schläger et al., 2016).

A deeper transcriptional profile of immune cells using scRNA-seq has been applied to animal models of MS to gain greater resolution of cell identities and phenotypes within the CSF. A group led by Dr. Gerd Meyer zu Hörste employed 10x Genomics sequencing technology to characterize the immune cell landscape in different tissues during neuroinflammation (Schafflick et al., 2021). Again, the rat was used as a model organism in order to obtain sufficient cells from the CSF, in this case 100–120 μL per animal. Pooled CSF samples from dozens of animals eventually provided sufficient cell numbers for sequencing (Heming et al., 2022). Interestingly, naive rats contained proportionally more CD4 T cells than blood, CNS parenchyma, or border tissues both by flow cytometric as well as scRNA-seq assessment. While a roughly five-fold expansion of CD4 T cells within the CSF was observed during EAE, further characterization of the T cell phenotype in the CSF was not undertaken. Rather, attention was drawn to the surprisingly large proportion of B cells found in the dura mater which unexpectedly contained immature subsets. These observations fit with concurrent reports implicating the meninges as a tissue-specific niche for local hematopoietic development and tolerance induction (Brioschi et al., 2021; Cugurra et al., 2021). Notably, the frequency of B cells in the CSF of naïve rats did not significantly change upon induction of EAE, remaining low. Blending experimentation in rats with typical active EAE induced in C57BL/6 mice, the Meyer zu Hörste group observed phenotypic changes in meningeal B cells during disease including reduced proliferation, maturation, and promotion of antigen presention (Schafflick et al., 2021). The dramatic dynamics of meningeal B cells contrasting with the unwavering paucity of B cells in the CSF observed during neuroinflammation raises questions regarding the dependence - or lack thereof – between border immunity and B cell trafficking in the CSF. While it is likely that CSF can mediate inflammatory effects of B cells (Lisak et al., 2012; Schropp et al., 2023), particularly in chronic disease (Mitsdoerffer and Peters, 2016), whether these changes are reflected by B cells circulating in the CSF or by soluble mediators alone remains to be determined. Clearly the ability to test hypotheses related to the requirement of CSF in meningeal B cell immunity and tissue residence using animal models is limited because of scant B cell numbers within the fluid compartment of the CNS. Nevertheless, these cutting edge animal model studies reflect a conceptual advance in contemplating the contribution of B cells in the pathogenesis of MS.

With the shift in focus away from T cells toward B cell pathophysiology and therapies in MS (Franciotta et al., 2008), experimentation using various B cell-dependent animal models has generated insights into mechanisms of CNS inflammation. For instance, ELT development and disruption has been explored successfully in multiple murine models (Molnarfi et al., 2013; Dang et al., 2015; Häusler et al., 2018; Parker Harp et al., 2019; Brand et al., 2021). Yet as noted, assessments of CSF B cells to inflammatory and therapeutic responses within the CNS during EAE and other models of MS have been limited. Indirect evaluation of CSF B cells *via* quantification and analysis of intrathecal antibodies in animal models has instead served as a surrogate for CSF B cells. Pursuit of immunoglobulin abundance and specificity within the CSF of animals with inflammatory demyelination of the CNS has taken place since the identification of OCBs as a biomarker of MS. Regrettably, a consensus amongst various MS animal models regarding OCBs does not exist. Oligoclonal IgG bands synthesized in the CNS compartment are present in rats with EAE (Rostrom et al., 2004). More commonly, identical banding patterns of immunoglobulins are present in both the CSF and serum, which occurred in guinea pigs, SJL mice, and rabbits with EAE (Glynn et al., 1982; Whitacre et al., 1982; Franciotta et al., 2008), although a commonly used relapsing EAE system in SJL mice exhibited discernable elevation in the CSF IgG index (Gilli et al., 2019). Further investigations into the source and antigenic targets of plasmablasts and plasma cells have been illuminating (Rojas et al., 2019; Pröbstel et al., 2020) and offer additional opportunities to define the contribution of B cells to neuroinflammation in animal models of MS.

Additional studies using viral models of MS in mice have incorporated CSF analyses. Murine models of MS involving *Coronaviridae* [Mouse hepatitis virus (MHV)] and *Picornaviridae* [Theiler’s murine encephalomyelitis virus (TMEV)] have been utilized to explore pathogenic mechanisms and therapies for CNS inflammatory demyelinating diseases (Libbey and Fujinami, 2021). Mice infected with the strain JHM of MHV (MHV-JHM) were used as an early demonstration of murine CSF acquisition methodology and utility of viral models of MS (Fleming et al., 1983). Cytologic analyses of 5–15 μL of CSF revealed a pleocytosis present only in mice with clinical impairment that consisted of similar proportions of T cells, B cells, and monocytes. Subsequent studies demonstrated an accumulation of virus-specific immunoglobulin within 100–200 μL of CSF of rats infected as neonates with MHV-JHM (Sorensen et al., 1984). While advanced flow cytometric profiles of CSF from rodents infected with MHV have not been reported, proliferating B cells were found within border tissues by immunohistochemistry in mice with chronic inflammatory demyelination, although as disease unfolded isotype-switched B cells tended to congregate in the parenchyma (DiSano et al., 2017), presumably recruited from the periphery in order to constrain viral replication and infectious spread (Marques et al., 2011). An immune-mediated, chronic inflammatory demyelinating disease also can be induced in mice after infection with TMEV. Studies on the role of B cells in TMEV-mediated disease have suggested a clonal expansion of B cells within the CNS. Analyzing an average of 8–10 μL of CSF per mouse, high levels of IgG were found within the CSF in accordance with abundant B cells observed within the meningeal and perivascular spaces of the spinal cord, but in the absence of appreciable blood–brain barrier disruption (Pachner et al., 2011; DiSano et al., 2019). These viral systems represent powerful alternatives to autoimmune models of MS, yet also lack the in-depth profile of CSF B cells that would provide insights into compartmentalized inflammation, trafficking, and timing of disease activity during MS.

Important work emphasizing the contribution to MS by B cells has utilized primate models. In Japanese macaques (*Macaca fuscata*) that develop spontaneous encephalomyelitis, OCBs can be detected (Blair et al., 2016). A more popularized model of MS, using the species *Callithrix jacchus* (Kap et al., 2016) has been used to explore the role of B cells in MS (Kap et al., 2010, 2011). However, no definitive studies on CSF B cells in this EAE model have been reported, likely given the miniature stature of marmosets (adults weigh between 250 and 500 g) which precludes routine CSF analyses. In contrast, adult rhesus monkeys (*Macaca mulatta*) typically weigh over several kilograms, availing them to CSF studies. Indeed, more contemporary studies using EAE in rhesus monkeys include efforts to characterize the effects of disease-modifying therapy on leukocyte trafficking in the CNS. Analyzing leukocytes obtained *via* the cisterna magna (typically yielding 0.5 mL) using flow cytometry at disease onset demonstrated that the CSF infiltrate was composed primarily of T cells and monocytes as opposed to B cells (Haanstra et al., 2013), whereas anti-MOG IgG and IgM were localized to the CSF (Haanstra et al., 2015). While these studies did not include naïve animals, CSF leukocytes were likely elevated in comparison to normal ranges of basic immune cells in the Rhesus monkey defined by basic cytology (Hou et al., 1996). Again, however, perplexing variability amongst non-human primate models of MS exists, as cynomolgus monkeys (*Macaca fascicularis*) do not routinely harbor unique OCB within the CSF 4 weeks after immunization with spinal cord homogenate emulsified in CFA (Gallo et al., 1989). While studies of B cells in these higher order models of neuroinflammation add value by uniquely modeling MS, questions regarding intrathecal B cells and immunoglobulin remain incompletely answered. Overall, pursuing the role of B cells in non-human primate models of MS exemplifies the challenges stemming from the heterogeneous nature of animal models as well as incomplete quantitative measures of CSF immune cells.

Other animal systems for modeling MS exist but are not commonly used and have employed limited CSF assessment. For example, spinal fluid examination during canine EAE identified a conspicuous pleocytosis obtained from unmentioned quantities of CSF (Moon et al., 2015). An idiopathic, spontaneous CNS disease of canines, granulomatous meningo-encephalomyelitis (GME), shares some features with MS. In particular, recent work reveals substantial meningeal inflammation characterized by large collections of B cells resembling tertiary lymphoid structures in a variety of dog breeds with GME (Church et al., 2021). While CSF evaluation was performed diagnostically but not reported in this study, prior reports on GME have demonstrated a mononuclear pleocytosis (Lowrie et al., 2013). Potential correlates of meningeal inflammation and B cell accumulation within the CNS could be ideally addressed in a larger species such as canines. Additionally, unique work exploring the role of CSF in animal models of MS has included an evaluation of cell-derived microvesicles during EAE. Seen in naive humans without inflammatory neurologic diseases, CSF microvesicles were found to be significantly more abundant in MS patients with relapses compared to those in remission and their levels correlated with the number of active lesions identified by MRI (Verderio et al., 2012). Modeling this feature of neuroinflammation in rodents, pooled CSF samples from rats were analyzed and found to contain microvesicles expressing myeloid proteins such as CX3CR1. Similar to cellular infiltrates in the CSF during autoimmune neuroinflammation, CSF microvesicles correlated with disease severity over the course of murine EAE. Whether other cells besides microglia contribute to the CSF collection of microvesicles and whether they mediate cellular immune effects during MS remain to be determined. Overall, the diversity of CSF evaluation, both by use of distinct animal models as well as by evaluating different immune cellular components, could be of great benefit to pursuing pathogenic mechanisms and treatment responses for MS.

## 3. Future aspects and additional considerations

The examination and characterization of CSF immune cells in various animal models is still quite insufficient relative to its potential for shedding light on the immune mechanisms of neuroinflammation in MS. Clearly, overcoming limited sample volumes obtained from different animals is essential. As shown recently in an elegant study exploring the role of alternatively activated neutrophils in neuroinflammation, diminutive anatomic tissues can still serve as a viable source of immune cells (Sas et al., 2020). Whether use of different species, surgical advancements, exploitation of different timing or anatomic routes of CSF sampling, or a combination thereof could be sufficient to surmount this barrier should be determined. Additionally, flow cytometric bar-coding to pool samples, such as during different stages of disease longitudinally or between diseases altogether, offers the ability to analyze small batches of cells in a merged collection *post-hoc*. This would mitigate some of the negative consequences from pooling of specimens, including contamination from one or more samples and/or blunting of biological variability (Férard, 1995; Schisterman and Vexler, 2008). Barcoding is integral to scRNA-seq, which is expected to be used extensively in future CSF studies, both in humans as well as in animal models of MS. Although with various limitations (Chen et al., 2019), transcriptomic profiles through techniques like scRNA-seq will make small sampling tenable. Even more detailed analyses from scRNA-seq facilitates are possible, such as antigen receptor sequencing. Quantification of TCR and B cell receptor clones from the CSF of various animal models of MS, particularly in the context of meningeal inflammation and temporal dynamics, will be in line with current studies in MS (Pappalardo et al., 2020; Ramesh et al., 2020) and could be tremendously useful in detailing mechanisms of adaptive immune responses difficult to ascertain from patients. In terms of CSF myeloid cell studies, the dearth of experimentation on animal CSF in models of MS represents a major gap in knowledge. Future investigation of myeloid cells and their function within the CSF compartment during neuroinflammation is very likely to contribute to concrete understanding of discrete roles for various BAMs and microglia that could benefit from modeling in animals. Addressing questions such as regionality of immune cell trafficking within the CSF and regulation of CSF flow by various immune cells within the cerebral and spinal fluid compartment in animal models of CNS inflammation could be highly valuable to MS and other neuroinflammatory conditions. Finally, using animal models to integrate cellular CSF characteristics with proteomics, CNS architecture, and tissue integrity, are obvious studies to undertake so that a more comprehensive viewpoint of inflammatory changes occurring in MS can be discerned. Hence, future studies involving CSF from various animal models of MS are rife with opportunity to parallel ongoing and future patient research and advance the understanding of MS causes and treatments.

## 4. Conclusion

Cellular characterization of the CSF is important to capturing unique features of inflammatory demyelination of the CNS and a complete understanding of the nature of MS. High dimensional CSF studies have not been fully leveraged in animal models of MS, which limits the utility of modeling altogether. Given access from the compartment adjacent to the tissue injured by immune responses, CSF not only harbors key immune ingredients, but likely mirrors the events occurring in the CNS borders and parenchyma during disease. Although challenges persist, efforts to build upon current data are deemed worthwhile and several current and emerging opportunities are available for optimal analysis of CSF from animal models of MS. Future CSF studies exploring similarities and differences between models will clarify the utility of each model system and ultimately lead to translational contributions from animal modeling of MS.

## Author contributions

GFW is solely responsible for the content of this manuscript.

## Funding

This work was supported in part by Merit Award # I01CX002383 from the United States Department of Veterans Affairs Clinical Sciences Research and Development Service. GFW was also supported by R21 NS121818 from the NINDS.

## Conflict of interest

The author declares that the research was conducted in the absence of any commercial or financial relationships that could be construed as a potential conflict of interest.

The reviewer OS declared a past co-authorship with the author GFW to the handling editor.

## Publisher’s note

All claims expressed in this article are solely those of the authors and do not necessarily represent those of their affiliated organizations, or those of the publisher, the editors and the reviewers. Any product that may be evaluated in this article, or claim that may be made by its manufacturer, is not guaranteed or endorsed by the publisher.
